# Comparison of contrast-enhanced MRI features of the (teno)synovium in the wrist of patients with juvenile idiopathic arthritis and pediatric controls

**DOI:** 10.1007/s00296-021-05041-9

**Published:** 2021-11-22

**Authors:** Jeffrey M. A. van der Krogt, F. Verkuil, E. Charlotte van Gulik, Robert Hemke, J. Merlijn van den Berg, Dieneke Schonenberg-Meinema, Angelika Kindermann, Koert M. Dolman, Marc A. Benninga, Taco W. Kuijpers, Mario Maas, Charlotte M. Nusman

**Affiliations:** 1 Department of Radiology and Nuclear Medicine, Amsterdam University Medical Centers (Amsterdam UMC), Location AMC, University of Amsterdam, Meibergdreef 9, 1105 AZ Amsterdam, The Netherlands; 2 Department of Pediatric Immunology, Rheumatology and Infectious Diseases, Emma Children’s Hospital, Amsterdam University Medical Centers (Amsterdam UMC), University of Amsterdam, Meibergdreef 9, 1105 AZ Amsterdam, The Netherlands; 3 Department of Pediatric Gastroenterology and Nutrition, Emma Children’s Hospital, Amsterdam University Medical Centers (Amsterdam UMC), University of Amsterdam, Meibergdreef 9, 1105 AZ Amsterdam, The Netherlands; 4 Department of Pediatrics, OLVG Hospital, Location West, Jan Tooropstraat 164, 1061 AE Amsterdam, The Netherlands

**Keywords:** Juvenile idiopathic arthritis, Magnetic resonance imaging, Wrist

## Abstract

To directly compare and describe the differences between juvenile idiopathic arthritis (JIA) patients and pediatric controls regarding features of the synovial and tenosynovial membrane on contrast-enhanced magnetic resonance imaging (MRI) of the wrist. T1-weighted contrast-enhanced MRI scans of 25 JIA patients with clinically active wrist arthritis and 25 children without a history of joint complaints nor any clinical signs of joint inflammation were evaluated by two readers blinded to clinical data. The synovium was scored at five anatomical sites based on thickening of the synovium (0–3 scale) and synovial enhancement (0–2 scale). Thickening and/or enhancement of the tenosynovium was scored at four anatomical sites using a 0–3 scale. Significantly higher scores for synovial thickening (median 4 vs. 1, *p* < 0.001) and synovial enhancement (median 4 vs. 1, *p* < 0.001) are found in the wrist of JIA patients as compared to controls. JIA patients experienced the highest synovial scores at the mid-/inter-carpal, 2nd –5th carpometacarpal, and radiocarpal joints. No significant difference in tenosynovial scores is found between both groups (median 0 vs. 0, *p* = 0.220). This study highlights the higher synovial thickening/enhancement scores on contrast-enhanced MRI of the wrist in JIA patients compared to pediatric controls. Tenosynovial thickening and/or enhancement was rarely present in both groups. In JIA patients, synovial thickening and enhancement were particularly present at three anatomical sites. These results substantially support rheumatologists and radiologists when navigating through MRI of the wrist in search for JIA disease activity.

## Introduction

Juvenile idiopathic arthritis (JIA) is typically characterized by soft tissue inflammation, such as synovitis and tenosynovitis [1]. At first presentation of JIA, disease activity in the wrist is present in 23% of all patients [2]. Contrast-enhanced magnetic resonance imaging (MRI) is a helpful technique for diagnosing and grading soft tissue pathology [3].

To reliably determine JIA disease activity in the wrist joint of children, contrast-enhanced MRI scores for the assessment of the synovium [4] and tenosynovium [5] in the wrist of children have been developed. Recently, a study on MRI findings in the wrist of children showed that mild (teno)synovial enhancement and/or thickening can be considered as normal [6]. Besides, a distribution pattern of preferred locations for disease activity in the JIA wrist has previously been established [7]. Direct comparison of the (teno)synovial MRI scores between JIA patients and pediatric controls has not yet been done. This knowledge contributes to a roadmap for the rheumatologist and radiologist to enable rapid and easy navigation through contrast-enhanced MRI of the wrist when assessing JIA disease activity.

The aim of this study is to directly compare and describe the differences between JIA patients and pediatric controls regarding features of the synovial and tenosynovial membrane on contrast-enhanced MRI of the wrist. Second, the distribution pattern of contrast-enhanced MRI features of the synovium and tenosynovium of the wrist is evaluated and compared between both groups.

## Materials and methods

### Clinically active JIA patients

JIA patients were selected from a multicenter prospective observational JIA patient database (May 2012–July 2013). Since a prolonged time interval between intravenous contrast agent administration and image acquisition is known to increase synovial thickness upon contrast-enhanced MRI examination [8], only JIA patients of whom the time interval from contrast fluid injection to the start of MRI examination was under 20 min were selected. All JIA patients had clinical arthritis in the wrist examined. JIA disease activity was scored by the referring pediatric rheumatologist using the Juvenile Arthritis Disease Activity Score-10 (JADAS-10) [9]. In addition, clinical disease remission or inactivity was ruled out using the Wallace criteria [10].

### Pediatric control group

Because of ethical objections, it is not possible to undergo a MRI procedure nor administer intravenous contrast agent to healthy children. Alternatively, children with suspected or confirmed inflammatory bowel disease (IBD), who were scheduled for IBD-related MR enterography with intravenous contrast agent administration, were prospectively included between July 2012 and March 2014. A similar approach was applied to a previous study on the comparison of enhancing synovial thickness in the knee upon contrast-enhanced MRI between clinically active JIA patients and pediatric controls [11]. Joint abnormalities, joint complaints and joint inflammation were ruled out in these children by a research fellow following the pediatric Gait Arms Legs Spine (pGALS) screening method [12]. The research fellow was trained to perform examination and the screening method by a pediatric rheumatologist (25 years of experience).

### MRI protocol

Clinically active JIA patients underwent an axial wrist T1-weighted MRI sequence with fat saturation (TR 400–750 ms, TE 10 ms; slice thickness 4 mm; field of view 150 × 150 mm, matrix 384 × 384) using a 1.0 T MRI scanner (Panorama HFO, Philips Healthcare), after intravenous contrast agent administration (Gadovist, Bayer Schering Pharma, Berlin, Germany, 1.0 mmol gadolinium/mL, dose 0.1 mmol/kg).

Children from the control group initially underwent MR enterography using a 1.5 T MRI scanner (MAGNETOM Avanto™, Siemens Medical Systems) after intravenous contrast administration (Gadovist, Bayer Schering Pharma, Berlin, Germany, 1.0 mmol gadolinium/mL, dose 0.1 mmol/kg). Following a change of position for correct placement of the wrist coil and without repeated intravenous contrast agent administration, an axial contrast-enhanced MRI sequence with fat saturation (TR 400–750 ms, TE 10 ms; slice thickness 4 mm; field of view 150 × 150 mm, matrix 384 × 384) was obtained from the wrist. Precautionary measures were made to ensure minimal time interval between intravenous contrast injection and image acquisition.

### Image analysis

**Fig. 1:**
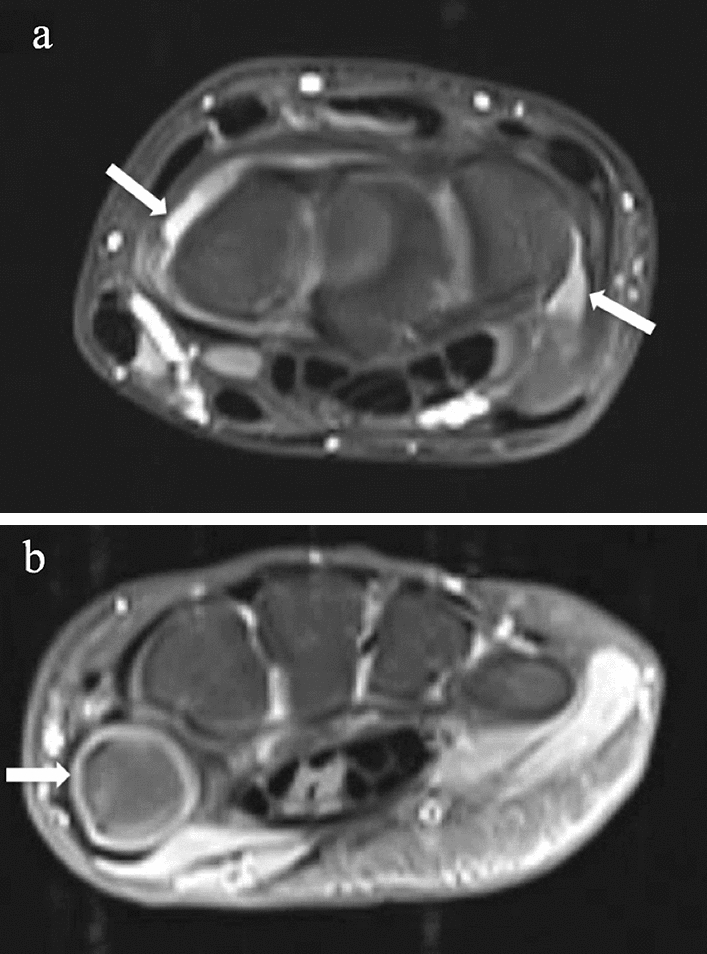
Hyper-intense cartilage mimics synovial enhancement. T1-weighted contrast-enhanced and fat-saturated MRI of **a** moderately increased synovial enhancement in the wrist of a 15-year female patient indicated by the arrows and **b** hyper-intense cartilage of the wrist in a 10-year old female patient indicated by the arrow

General agreement on the scores and conformity on the appearance of hyper-intense structures were achieved during a preliminary calibration session (Fig. 1). After being blinded for clinical data, image sets of each participant were scored by two musculoskeletal radiologists (12 and 25 years of experience in musculoskeletal radiology) by means of consensus. Two MRI features of the synovium and one of the tenosynovium were evaluated according to existing scoring methods [4, 5].

**Fig. 2:**
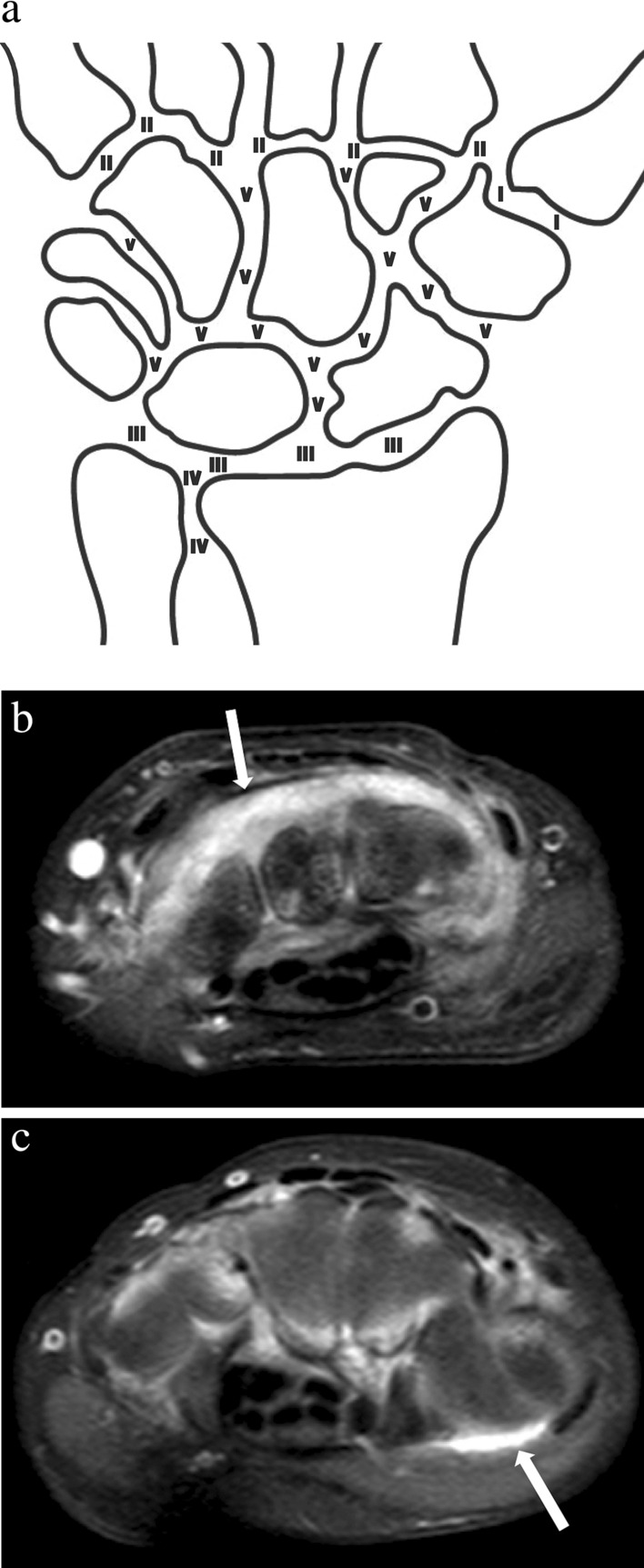
**a** Anatomical scoring sites of the synovium. (I) First carpometacarpal recess, (II) 2nd–5th carpometacarpal joints, (III) Radiocarpal joint, (IV) Distal radioulnar joint, (V) Mid-/inter-carpal joints. Example of a contrast-enhanced MRI image of **b** severe synovial thickening in the wrist of a 17-year-old female JIA patient, and (**c**) severe synovial enhancement in the wrist of a 9-year-old female JIA patient

In accordance with the scoring method introduced by Damasio et al. [4], the synovium was assessed for two features, namely thickening of the synovium and synovial enhancement. Effusion was out of the scope of this study, since no T2-weighted sequences were available. In the current study, both synovial features were scored at five predefined anatomical sites: (1) carpometacarpal recess 1, (2) carpometacarpal joints 2–5, (3) radiocarpal, (4) distal radioulnar, and (5) mid-/inter-carpal (Fig. 2a). First, the degree of thickening of the synovium was scored (0: no thickening, 1: mild thickening, 2: moderate thickening, 3: severe thickening). An example of a contrast-enhanced MRI image of severe thickening in the wrist of a 17-year-old female JIA patient is displayed in Figure 2b. Second, the degree of synovial enhancement was scored (0: normal synovial enhancement as compared to neighboring muscle, (1) mildly increased synovial enhancement, (2) moderately to severely increased synovial enhancement. An example of a contrast-enhanced MRI image of severely increased synovial enhancement in the wrist of a 9-year-old female JIA patient is displayed in Figure 2c. Per individual child, total scores of both synovial features were separately obtained by summing the scores of each feature from all five anatomical scoring sites, generating maximum achievable total scores of 15 for thickening of the synovium and 10 for synovial enhancement.

**Fig. 3:**
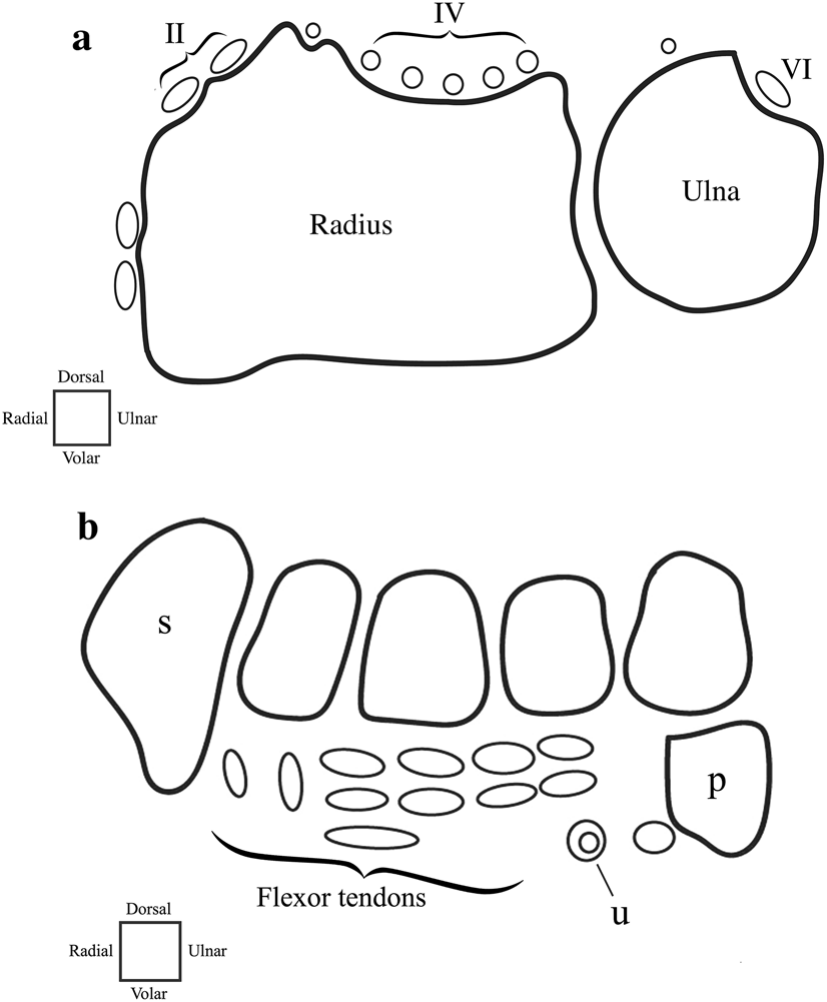
**a** Tenosynovium extensor tendon scoring compartments (II) extensor carpi radialis longus/extensor carpi radialis brevis, (IV) extensor indicis proprius/extensor digitorum communis, (VI) extensor carpi ulnaris, (LT) Lister’s tubercle and (**b**) flexor tendon scoring compartment (s) scaphoid, (p) pisiform, (u) ulnar artery

In accordance with the scoring method introduced by Lambot et al. [5], the tenosynovium was assessed by a semi-quantitative simultaneous evaluation of enhancement and thickening of the tendon sheath (0: no enhancement and no thickened synovial sheath, (1) enhancement and mildly thickened synovial sheath, (2) enhancement and moderately to significantly thickened synovial sheath). The tenosynovium was evaluated in both extensor and flexor tendons. Extensor tendons (second, fourth, and sixth compartments) were scored at the axial height of Lister’s tubercle, as illustrated in Fig. 3a. Flexor tendons were scored as a bundle at the axial height of the carpal tunnel, as illustrated in Fig. 3b. Per individual child, the total score for tenosynovial thickening and enhancement was obtained by summing the scores from all four anatomical scoring sites, generating a maximum achievable total score of 8.

Since the originally proposed scoring methods for the assessment of the (teno)synovium to which we refer in the current study proved intra-observer agreements sufficient for clinical use [4, 5], participants of the current study were scored once, in consensus between both readers.

### Statistical analysis

Based on a previous study comparing contrast-enhanced MRI features of the (teno)synovium in the knee of JIA patients with pediatric controls [11], we made a sample size calculation in nQuery based on comparing means with a Mann–Whitney *U* test for continuous outcomes with the following numbers: alpha 0.05, 2-sided, power 80%, mean of 0.92 in the control group, 2.92 in the JIA group and a common standard deviation of 2.329. This resulted in a number of 26 patients per study group, similar to our presented work. The Shapiro–Wilk test was used to test for normality of the results within study groups. Descriptive statistics were used to specify baseline characteristics in terms of number (percentage), mean [standard deviation (SD)], and median [interquartile range (IQ-r)]. Unpaired t tests were used to examine differences in time interval from contrast fluid injection to start MRI between the group of JIA patients and the pediatric control group. Mann–Whitney *U* tests were performed to analyze differences in age and (teno)synovial scores between both groups. Statistical significance was defined as a *p* value < 0.05. These statistical analyses were performed with the use of IBM SPSS Statistics, Version 26 (IBM Corporation).

### Compliance with ethical standards

The authors declare that there is no conflict of interest. This study has been conducted according to the World Medical Association Declaration of Helsinki. Institutional review board approval by the Medical Ethics Committee at the XXX in XXX was obtained (XXX). Written informed consent to participate and for publication was obtained from all individuals included.

## Results

### Participant characteristics

Twenty-five clinically active JIA patients (68% female) and 25 children from the control group (44% female) were included. On average, children from the control group were significantly older (mean ± SD 14.9 ± 2.3 vs. 13.2 ± 2.8 years, *p* < 0.05) and had a significantly longer time interval from intravenous contrast injection to image acquisition as compared to JIA patients (median ± IQ-r 11.5 ± 5 vs. 10.0 ± 1 min, *p* < 0.001). Complete baseline characteristics of the children included are summarized in Table 1.

**Table 1:** Baseline clinical characteristics of study groups

	Clinically active JIA patients (*n* = 25)	Control group (*n* = 25)
No. (%) of female subjects	17 (68)	11 (44)
Age at MRI, years; mean (SD)*	13.2 (2.8)	14.9 (2.3)
Time interval from intravenous contrast to MRI wrist, minutes; median (IQ-r)***	10 (1)	11.50 (5)^a^
JADAS-10 (0–40), median (IQ-r)	7.5 (12)	–
Diagnosis JIA, no. (%)		
Oligo-articular persistent	5 (20)	–
Oligo-articular extended	3 (12)	–
Poly-articular RF-	11 (44)	–
Poly-articular RF +	1 (4)	–
Psoriatic arthritis	2 (8)	–
Enthesitis related JIA	2 (8)	–
Unclassified	1 (4)	–
PCDAI, *n* = 18; mean (SD)	–	23 (14)
PUCAI, *n* = 7; mean (SD)	–	35 (16)
Diagnosis IBD^b^, no. (%)		
Crohn’s disease	–	14 (56)
Ulcerative colitis	–	7 (28)
No or other disorder	–	4 (16)

Values are mean (standard deviation) for age, PCDAI and PUCAI. Values are median (interquartile range) for time interval and JADAS-10

*MRI* magnetic resonance imaging, *PCDAI* Pediatric Crohn’s Disease Activity Index, *PUCAI* Pediatric Ulcerative Colitis Activity Index, *IBD* inflammatory bowel disease, *JIA* Juvenile idiopathic arthritis, *JADAS* Juvenile Arthritis Disease Activity Score, *RF* rheumatoid factor

*p ≤ 0.05, ***p ≤ 0.001, ^a^*n* = 24, ^b^final diagnosis

### Total scores of the synovium and tenosynovium

**Fig. 4:**
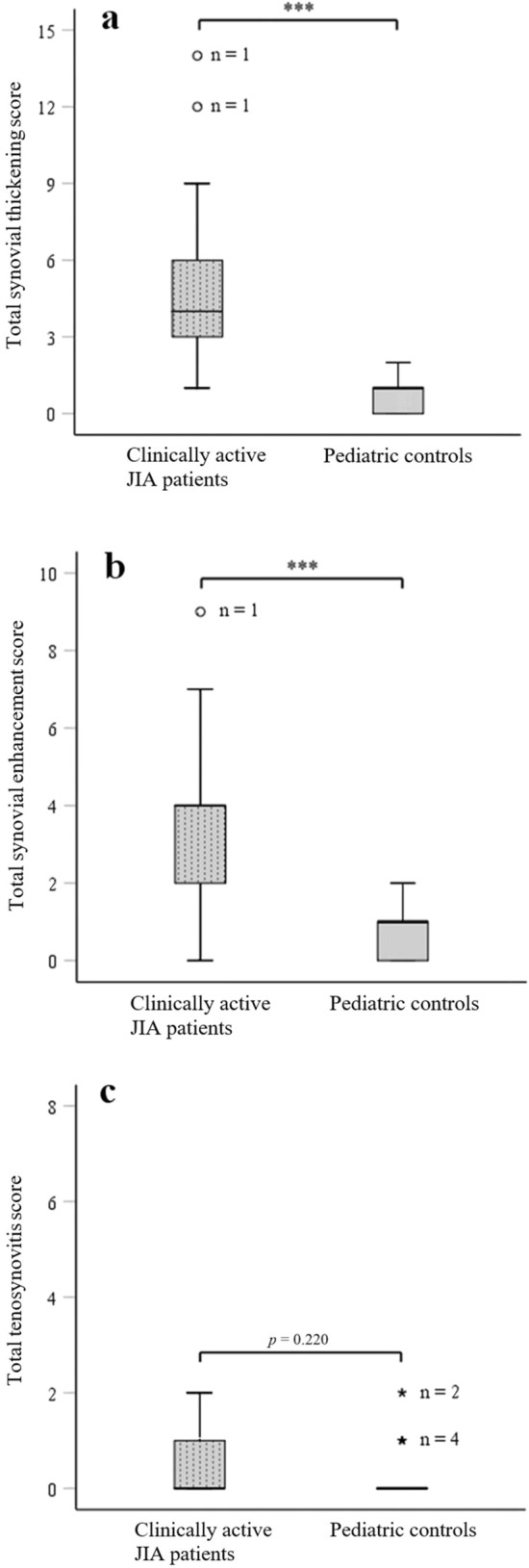
Differences in total synovial and total tenosynovitis scores. Boxplot visualizations of total scores for **a** synovial thickening, **b** synovial enhancement, and **c** tenosynovial thickening/enhancement in clinically active JIA patients (left, dotted boxes) and pediatric controls (right, open boxes). ***p < 0.001, [o]: statistical outlier, “Asterisk” extreme statistical outlier

Within the group of clinically active JIA patients, both the total synovial thickening score (median ± IQ-r 4 ± 3 vs. 1 ± 1, *p* < 0.001; Fig. 4a) as well as the total synovial enhancement score (median ± IQ-r 4 ± 2 vs. 1 ± 1, *p* < 0.001; Fig. 4b) appeared significantly higher as compared to the control group. Regarding the total tenosynovial thickening and/or enhancement score, no significant difference was found between clinically active JIA patients and controls (median ± IQ-r 0 ± 1 vs. 0 ± 1, *p* = 0.220; Fig. 4c).

### JIA-specific distribution pattern of (teno)synovial features

**Fig. 5:**
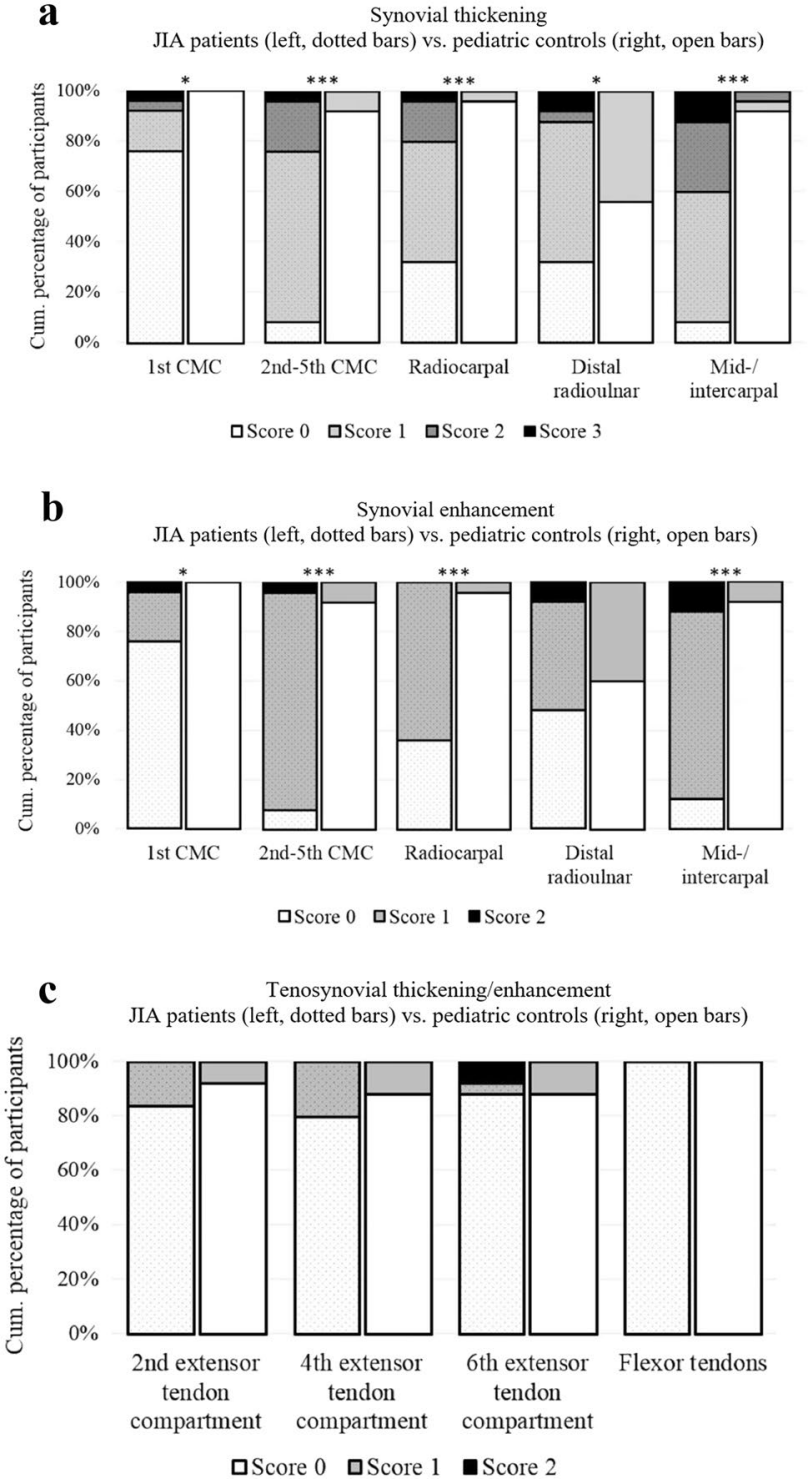
For both the JIA patients (left, dotted bars) and the pediatric controls (right, open bars), the divisions of scores for **a** synovial thickening, **b** synovial enhancement, and **c** tenosynovial thickening/enhancement. *p < 0.05, ***p < 0.001

To determine the JIA-specific distribution pattern of (teno)synovial features in the wrist on contrast-enhanced MRI, scores from all separate anatomical scoring sites were compared between JIA patient and control groups. In comparison to pediatric controls, clinically active JIA patients showed significantly higher scores of synovial thickening at all five anatomical scoring sites (Fig. 5a). Regarding synovial enhancement, scores were significantly increased in JIA patients at the first carpometacarpal recess, 2nd–5th carpometacarpal, mid-/inter-carpal and radiocarpal joints (Fig. 5b). Further evaluation of the distribution pattern of tenosynovial thickening and/or enhancement showed no significant differences between both groups at any of the four anatomical scoring sites (Fig. 5c).

**Fig. 6:**
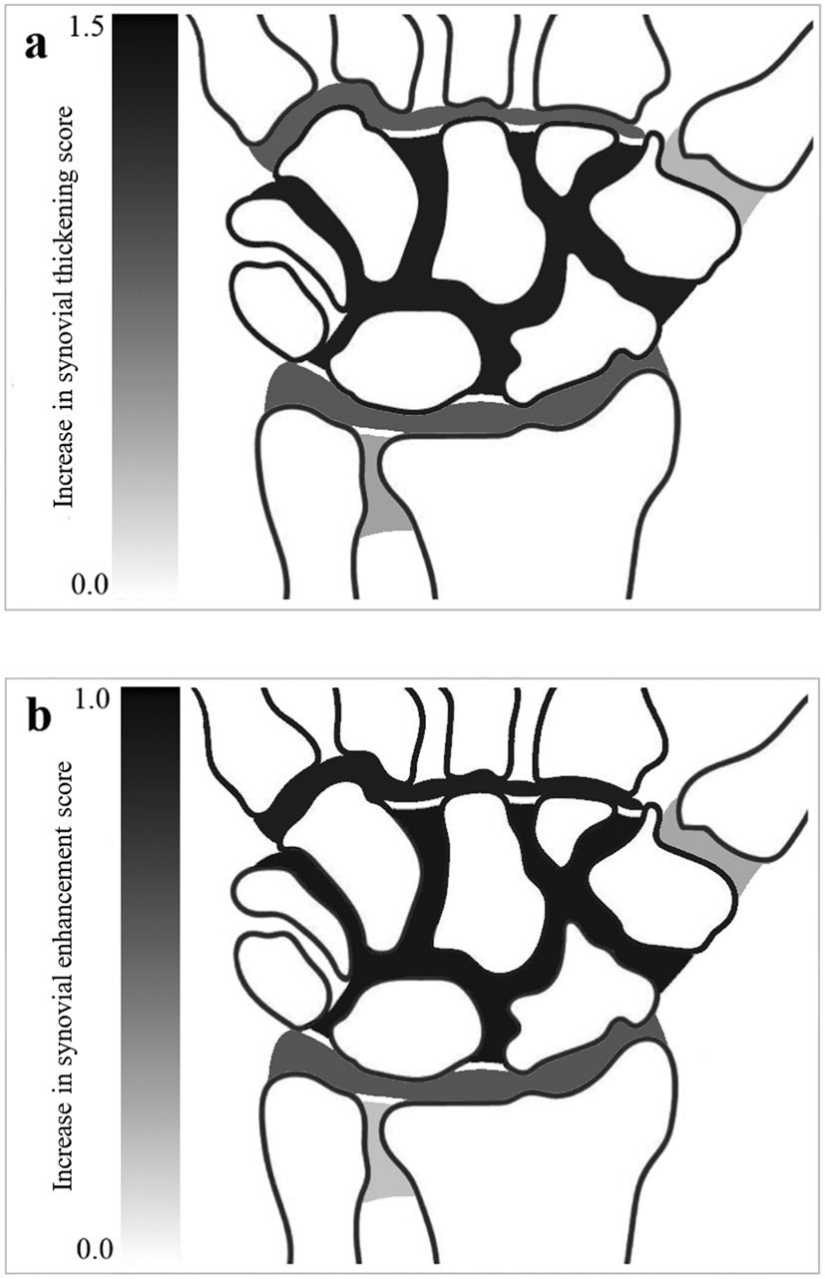
Grayscale map illustrating darker areas more likely to reflect JIA disease activity than normal variance. Based on a grayscale gradient, the increase in mean score within the wrist of clinically active JIA patients, as compared to controls, have been visualized for **a** synovial thickening, **b** synovial enhancement. In this figure, darker areas indicates the highest increase in total score, whereas lighter areas indicates no increase in total score

A grayscale map has been created to visualize the difference in scores for both clinical features of synovium in the wrist between JIA patient and control groups (Fig. 6). This map indicates three anatomical scoring sites to be major determinants for the difference in synovial scores between JIA patients and controls, namely the mid-/inter-carpal, the 2nd–5th carpometacarpal, and the radiocarpal joints.

## Discussion

This is the first study to directly compare contrast-enhanced MRI features of the synovium and tenosynovium in the wrist between clinically active JIA patients and pediatric controls. Synovial thickening and -enhancement, was significantly more present in JIA patients compared to pediatric controls, in whom it was present in a low grade. Based on the results of this study, the mid-/inter-carpal, carpometacarpal 2nd–5th, and radiocarpal joints are susceptible for developing JIA disease activity. No difference in the tenosynovial score was found between the two groups.

Separate analysis of anatomical scoring sites for synovial thickening and synovial enhancement revealed the carpometacarpal recesses of digits 2–5, the mid-/inter-carpal, the radiocarpal, and the distal radioulnar joints to be highly affected in the wrist of clinically active JIA patients. On the contrary, the first carpometacarpal recess generated low scores for synovial thickening in JIA patients. A previous study on JIA disease activity, without comparison to controls, revealed the distal radioulnar joint as one of the top three locations affected by synovial thickening in the wrist of JIA patients [7]. Although the results of the current study are comparable to that finding, our study additionally indicates synovial thickening to be also frequently detected in the distal radioulnar joint of pediatric controls. This finding is in line with a previous study on the appearance of MRI findings in the wrist of inflammatory-free children, in which the distal radioulnar was the only anatomical scoring site on which moderate–severe enhancement of the synovium was scored [6].

Low scores for tenosynovial thickening/enhancement were found within the wrist of clinically active JIA patients. At first, this result seems contradictive to the results published by a study on the distribution pattern of MRI abnormalities within the joints of JIA patients, which showed presence of wrist tenosynovitis in 46.5% of JIA patients investigated [7]. However, in that study, the severity of the tenosynovial thickening/enhancement was not taken into account, which might have led to an overestimation of the contribution of tenosynovial thickening/enhancement to overall wrist pathology in JIA patients. In comparison, a same approach would have generated a presence of tenosynovial thickening/enhancement in 44% of JIA patients in the current study, which is consistent with the previous findings. Within the control group, tenosynovial thickening/enhancement scores were also found to be low, resulting in the absence of a difference in tenosynovial score among clinically active JIA patients as compared to controls.

This study encountered several limitations. First, the establishment of a pediatric healthy control group was not possible since it is undesirable to undergo MRI and administer intravenous contrast agent to healthy children. Due to a reported prevalence of clinical arthritis in 8–12% of the patients diagnosed with IBD, the underlying disease of in the largest part these pediatric controls is suboptimal with respect to the objective of the study [13, 14]. Although our pediatric controls in this study were the best available control population, and precautionary measures were taken to rule out arthritis in this group, follow-up studies should aim to include a non-inflammatory control group and acquire a larger study population. Second, the controls had a significantly longer time interval from intravenous contrast administration to image acquisition as compared to JIA patients. Given the positive correlation found between post-gadolinium time interval and synovial enhancement in the wrist of children on contrast-enhanced MRI [15], this difference in post-contrast timing might have led to an overestimation of synovial thickening and synovial enhancement in controls. A third limitation comprised the different MRI field strengths that were applied to the two groups of children (1.5 T vs. 1.0 T, respectively), which might have influenced the assessment procedure. Together, these observations indicate the need to meticulously standardize MRI protocols with regard to (1) the time interval from intravenous contrast administration to image acquisition and (2) the applied MRI field strength. Lastly, the JIA patients in this study form a heterogenic group with respect to age and JIA subtype, but the group size was not sufficient to perform subgroup analyses.

In conclusion, this study adds to the knowledge on discriminative characteristics of (teno)synovium in the wrist of both JIA patients with active disease and controls. Furthermore, this study highlights the strong increase in synovial scores in clinically active JIA patients in comparison to controls at three anatomical scoring sites. These characteristic differences in the pattern of synovial thickening and enhancement in the wrist between both groups can be very helpful for rheumatologists and radiologists when navigating through MRI of the wrist in search of JIA disease activity. Further research should focus on comprehensive validation of the synovial thickening and enhancement scores by including non-inflammatory controls, acquiring a larger study population, and applying meticulous standardization to the MRI protocols.

## Declarations

### Data sharing

Authors are willing share data regarding our manuscript upon request.
